# Targeting cattle for malaria elimination: marked reduction of *Anopheles arabiensis* survival for over six months using a slow-release ivermectin implant formulation

**DOI:** 10.1186/s13071-018-2872-y

**Published:** 2018-05-04

**Authors:** Carlos J. Chaccour, Kija Ngha’bi, Gloria Abizanda, Angel Irigoyen Barrio, Azucena Aldaz, Fredros Okumu, Hannah Slater, Jose Luis Del Pozo, Gerry Killeen

**Affiliations:** 1 Instituto de Salud Tropical, Universidad de Navarra, Pamplona, Spain; 2 ISGlobal, Hospital Clínic, Universitat de Barcelona, Barcelona, Spain; 3 Environmental Health and Ecological Sciences Department, Ifakara Health Institute, Ifakara, United Republic of Tanzania; 4 School of Health Sciences, University of Dar es Salaam, Dar es Salaam, Tanzania; 5 Centro de Investigación Médica Aplicada, Pamplona, Spain; 6 Drug Development Unit Universidad de Navarra (DDUNAV), Pamplona, Spain; 7 Department of pharmacy, Clínica Universidad de Navarra, Pamplona, Spain; 8 School of Public Health, University of the Witwatersrand, Johannesburg, South Africa; 9 MRC Centre for Outbreak Analysis & Modelling, Department of Infectious Disease Epidemiology, Imperial College London, London, UK; 10 Department of Microbiology, Clínica Universidad de Navarra, Pamplona, Spain; 11 Infectious Diseases Division, Clínica Universidad de Navarra, Pamplona, Spain; 12 Department of Vector Biology, Liverpool School of Tropical Medicine, Liverpool, UK

**Keywords:** Residual transmission, Ivermectin, Endectocides, Cattle, Zoophagy, Slow release, Pharmacokinetics

## Abstract

**Background:**

Mosquitoes that feed on animals can survive and mediate residual transmission of malaria even after most humans have been protected with insecticidal bednets or indoor residual sprays. Ivermectin is a widely-used drug for treating parasites of humans and animals that is also insecticidal, killing mosquitoes that feed on treated subjects. Mass administration of ivermectin to livestock could be particularly useful for tackling residual malaria transmission by zoophagic vectors that evade human-centred approaches. Ivermectin comes from a different chemical class to active ingredients currently used to treat bednets or spray houses, so it also has potential for mitigating against emergence of insecticide resistance. However, the duration of insecticidal activity obtained with ivermectin is critical to its effectiveness and affordability.

**Results:**

A slow-release formulation for ivermectin was implanted into cattle, causing 40 weeks of increased mortality among *Anopheles arabiensis* that fed on them. For this zoophagic vector of residual malaria transmission across much of Africa, the proportion surviving three days after feeding (typical mean duration of a gonotrophic cycle in field populations) was approximately halved for 25 weeks.

**Conclusions:**

This implantable ivermectin formulation delivers stable and sustained insecticidal activity for approximately 6 months. Residual malaria transmission by zoophagic vectors could be suppressed by targeting livestock with this long-lasting formulation, which would be impractical or unacceptable for mass treatment of human populations.

**Electronic supplementary material:**

The online version of this article (10.1186/s13071-018-2872-y) contains supplementary material, which is available to authorized users.

## Background

Indoor, human-targeted vector control with long-lasting insecticidal nets (LLINs) and indoor residual spraying (IRS) accounts for most of the reductions in malaria burden since 2000 [1]. However, further progress is limited by residual transmission, mediated by mosquitoes that avoid LLINs/IRS by feeding and/or resting outdoors [2, 3]. Also, physiological resistance to the four insecticide classes approved for public health use threatens these gains [4]. In 2016 there were approximately 445,000 malaria-related deaths globally and an increase in cases from 211 to 216 million, representing a return to the 2012 levels [5]. The global fight against malaria is at a crossroads [6] and no longer on track to achieve the goal of the WHO Global Technical Strategy (GTS) of reducing cases by 90% and eliminating malaria from 35 countries by 2030 [6, 7]. New vector control approaches and active ingredients [8] are needed to both tackle residual transmission and mitigate against resistance, respectively [2–4]. Eliminating malaria in many settings will require reducing the biological coverage gap left by existing LLIN/IRS measures [9], by accelerating innovation and market entry of new approaches to malaria vector control [6, 10].

The primary mechanism of action of existing LLIN and IRS interventions is actually vector population suppression, rather than the more obvious personal protection they provide against mosquito bites [11]. LLINs and IRS are so effective in highly endemic parts of Africa and Oceania because the most efficient vectors in these regions feed consistently on humans, indoors and at night [12]. However, the large majority of vectors capable of transmitting malaria in various parts of the world feed primarily upon animals [11], but also occasionally upon humans [2, 11, 13]. Mosquito species that obtain even a small percentage of their blood meals from humans can mediate relatively low, but nevertheless self-sustaining, levels of transmission that respond poorly to human-targeted LLINs or IRS [11, 14–17]. However, an even more important behavioural category of vectors is those which feed readily, opportunistically and flexibly upon either animals or humans [11]. Vectors with such dual feeding preferences are ubiquitously associated with residual malaria transmission because they feed often enough on humans to mediate intense transmission, but also often enough on animals to survive and reproduce despite high coverage of LLINs and/or IRS [11]. Furthermore, feeding upon livestock is often associated with additional behaviors that allow avoidance of insecticides, such as feeding outdoors, at dusk/dawn or exiting houses quickly after feeding [2, 11]. Successful scale-up of human-centred indoor vector control with LLINs and IRS in recent years has had the greatest impact upon the most efficient, human-specialized vectors, so more zoophagic species exhibiting these advantageous behaviors now account for increasing proportions of persisting vector populations and residual transmission [18]. Furthermore, as humans are increasingly protected with LLINs and IRS, the phenotypically plastic behaviours of most mosquito species allows them to survive by making greater use of animal blood source [2, 17, 19]. New tools targeting partly zoophagic vectors may be needed to eliminate malaria in many settings where they contribute towards sustaining residual transmission [11, 15].

Ivermectin is an antiparasitic drug used for the control of onchocerciasis, lymphatic filariasis and other neglected tropical diseases (NTDs) in humans, as well as a wide array of endo- and ectoparasites in livestock and pets. Ivermectin is also an endectocide, meaning it also has systemic insecticidal properties when administered as a drug, shortening the lifespan of mosquitoes and other arthropods feeding on treated subjects [20]. If used at scale, ivermectin could potentially reduce malaria transmission [21, 22], by targeting malaria vectors regardless of place and time of biting, thus offering a complementary strategy to LLINs and IRS for malaria elimination. This has motivated a recent review by WHO [23] and publication of the preferred product characteristics (PPC) of endectocides for malaria transmission control [24].

The impact of an ivermectin-based strategy would be driven by (i) the proportion of blood sources (both human and animal) that are covered with the intervention [9]; (ii) the drug levels achieved in the blood of treated subjects [20, 25]; and (iii) the duration of time over which a single treatment achieved sufficient blood concentrations to kill mosquitoes [25, 26]. Of these parameters, duration of mosquito-killing concentrations is probably the most practically limiting for achieving sufficient affordability, effectiveness and population-wide coverage [27, 28].

Targeting livestock with endectocides offers two potential advantages: (i) improved transmission suppression through increased biological coverage of all the blood sources that are relevant to sustaining vector populations [9]; and (ii) it is possible to administer a wider diversity of veterinary formulations and high doses to animals in a manner than would not be acceptable for humans. In the case of ivermectin, there is proven efficacy against *Anopheles arabiensis* [29, 30] and veterinary application allows for more flexibility in dose, regimen or formulations compared with what would be practical in humans. We have optimized an implantable, slow-release ivermectin formulation [31, 32] for use in cattle and other livestock, as an improved tool for enabling more effective malaria vector control while also improving livestock health and economic productivity, as well as controlling multiple livestock-mediated zoonoses. Here we report the pharmacokinetic and entomological results obtained with this new formulation.

## Methods

### Experimental design

After basal blood sampling and mosquito feeding, three calves were randomly allocated to treatment or control; two were assigned to the ivermectin arm and received a dose of five subcutaneous implants and one untreated calve served as control. The dose was calculated based on the formulations excipient composition, elution surface and the calves’ expected weight for age. The control calf received no ivermectin. Fortnightly for 44 weeks, a group of *Anopheles arabiensis* was feed directly on the calves. Concurrently, blood was drawn to determine the ivermectin concentration present at the time of feeding (see Table 1 for timing of all procedures). Mosquito mortality was recorded daily for 10 days after feeding on the calves at every time point. All mosquito exposure experiments were performed in triplicate. The main outcome measures were mosquito mortality after 3 days and 10 days.

**Table 1:** Study procedures

Procedure	Weeks																						
	0	2	4	6	8	10	12	14	16	18	20	22	24	26	28	30	32	34	36	38	40	42	44
Basal blood sample	●	–	–	–	–	–	–	–	–	–	–	–	–	–	–	–	–	–	–	–	–	–	–
Basal mosquito feeding	●	–	–	–	–	–	–	–	–	–	–	–	–	–	–	–	–	–	–	–	–	–	–
Implantation	●	–	–	–	–	–	–	–	–	–	–	–	–	–	–	–	–	–	–	–	–	–	–
Daily aspect and behaviour check	●●●●●●●●●●●●●●●●●●●●●●●●●●●●●●●●●●●●●●●●●●●●●●●●●●●●●●●●●●●●●●●●●●●●●●●●●●●●●●●●●																						
Recurrent blood sampling	–	●	●	●	●	●	●	●	●	●	●	●	●	●	●	●	●	●	●	●	●	–	–
Recurrent mosquito feeding	–	●	●	●	●	●	●	●	●	●	●	●	●	●	●	●	●	●	●	●	●	●	●

### Ivermectin formulation

**Fig. 1:**
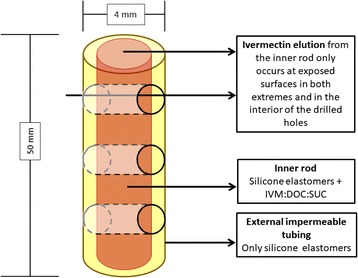
A schematic depiction of the formulation. *Abbreviations*: IVM, ivermectin; DOC, deoxycholate; SUC, sucrose

An optimized variation of a previously described silicone-based, slow release, subcutaneous formulation was used [31] (Fig. 1). In brief, extruded tubing of medical grade silicone with a 3.81 mm internal diameter and 0.25 mm thick wall (Freudenberg Medical, Carpinteria, CA, USA) was cut in 7 cm segments and filled with mixture of ivermectin, sodium deoxycholate, sucrose (all from Sigma-Aldrich, St. Louis, MO, USA) and unrestricted drug delivery silicone (DDU-4320, NuSil, Carpinteria, CA, USA) using a pneumatic dispenser. Drug powder, excipients and elastomers were mixed using the method described by Maeda et al. [33] and Cunningham et al. [34]. The tubes were then cured at 60 °C for 4 h and post-cured at room temperature for additional 18 h. The resulting products were then trimmed to 5 cm length. Once inserted, the drug-eluting inner rod is only exposed to subcutaneous tissue and fluids at the extremes of the formulation. There, the sucrose in the inner cylinder is slowly diluted, creating micro channels that allow for slow release of the drug.

Previous experiments have shown that the exposed area of the drug-eluting core is a key driver of systemic ivermectin levels [31, 32]. To increase release rate and achieve sufficient ivermectin levels in cattle, three 1 mm holes were drilled across each formulation. Each implant contained approximately 73 mg of ivermectin and had an elution surface of 95 mm^2^. After packaging, the rods were sterilized using an electron beam.

### Cattle procedures

Calves were aged 4 to 5 months when purchased from local farmers at the Kilombero valley (approximate weight at purchase 160 kg) at the outset of the study. They were kept free-ranging in the pastures surrounding the semi-field systems of the Ifakara Health Institute in Ifakara [35].

Each calf received five implants in the lateral surface of the neck by means of a single subcutaneous puncture with a 5 mm plastic trocar. Procedures were done under sterile conditions and using local anesthesia. All received a unique ear tag after treatment. Insertion wounds were treated topically with chlorhexidine. No calf received any systemic treatment during the experiment. Their general behavior was reviewed daily by a herdsman and in monthly visits by a qualified veterinarian.

Before implantation and at 2 weeks intervals until 40 weeks after treatment (21 time-points), the calves were mechanically restricted to draw 5 ml of blood from the jugular vein for high-performance liquid chromatography (HPLC) analysis. The mechanical restraint also served to feed a group of 150 *Anopheles arabiensis* (previously starved of sugar for 2 h) on every calf at every occasion until week 44 (23 time-points).

### Mosquito procedures

The mosquitoes used were *Anopheles arabiensis* from a colony stablished at the Ifakara Health Institute in 2014 by collecting wild specimens in nearby villages. The colony is kept inside the semi-field system at temperature and humidity that fluctuates naturally with the local climate [35].

**Fig. 2:**
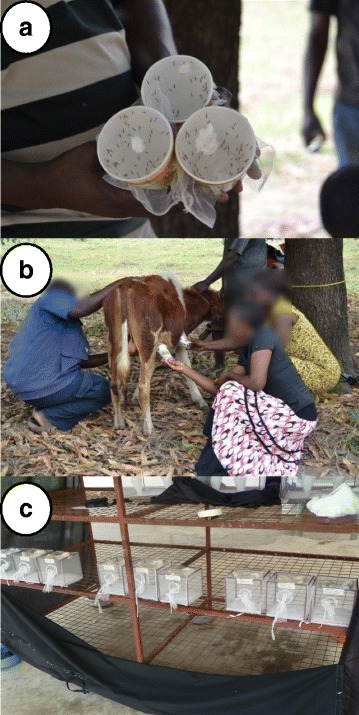
Mosquito feeding and follow-up procedures. **a** Triplicates of 50 mosquitoes fed on each calf. **b** Mosquitoes were fed by applying the cups to shaved areas in the abdomen until all or most were fully engorged. **c** Each group of 50 mosquitoes was then kept in individual cages and allowed to feed on water and sugar *ad libitum*, they were followed for 10 days and mortality recorded daily

Feeding assays were conducted every two weeks after implantation, before every assay, 2–3 days old hungry adult females were selected from the colony by holding an open palm next to the cage and gently aspirating those trying to bite. They were then transferred to paper cups (approximately 50 per cup) marked according to the assigned calf and starved of sugar for 2 h (Fig. 2a). A total of 150 (145–170) females were fed on each calf (triplicates of 50). The papers cups were covered with netting and applied to shaved areas of the calves’ abdomen for 30 min (Fig. 2b). Unfed and partly fed females were discarded and all fully engorged females from a single cup were then transferred to a mosquito cage for survival assessment, where they were allowed *ad libitum* access to water and 5% sucrose solution. The cages were kept on a shelf covered with a black cotton sheeting to protect it from any strong winds (Fig. 2c). Each cage was monitored daily for mosquito survival for 10 days and dead mosquitoes were recorded and removed from the cage. Following 10 days, mosquitoes remaining alive were killed by desiccation.

### Ivermectin quantification

Blood was drawn in 5 ml EDTA tubes, centrifuged and plasma aliquots were separated and frozen at -20 °C. Ivermectin was quantified using a previously described HPLC method [36]. The detection and quantification limits were 0.1 ng/ml and 0.5 ng/ml, respectively.

### Statistics

Mosquito survival analysis after feeding on the calves at each time point was accomplished using the Kaplan-Meier method, implemented with Addinsoft’s XLSTAT® Version 19.4.45479 (New York, USA). Comparisons of survival patterns were done with Log-rank test using a 5% significance level. For mosquitoes feedings at each time point, the proportions that survived for either 3 or 10 days (the approximate lengths of gonotrophic and sporogonic cycle, respectively) was calculated. Survival curves for each treatment at each time point were constructed to visualize variations over the study duration.

Additionally, individual hazard ratios (and 95% confidence intervals) for each treatment and feeding time point were calculated and fitted to a Cox’s proportional hazards model with ‘week post-implant’ as a covariate (fourth order polynomial function) to data from weeks 2 through to 44. A linear relationship was fitted to data from weeks 0 to 2.

## Results

### Pharmacokinetics

**Fig. 3:**
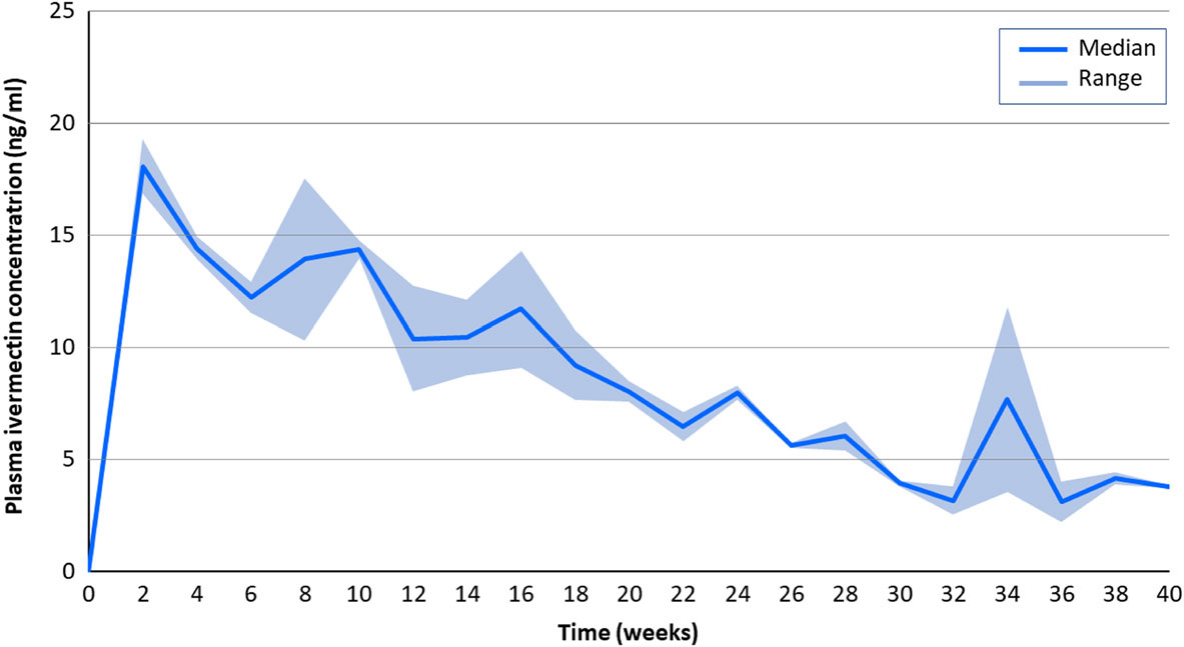
Ivermectin plasma levels sustained with the implant formulation adapted for cattle in this experiment

**Fig. 4:**
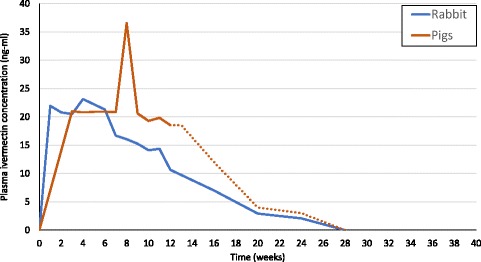
Ivermectin plasma levels sustained with previous versions of the same formulation adapted for 5 kg rabbits [31] and 80 kg pigs [32]. The dotted line in the pig results has been extrapolated based on the implants’ residual ivermectin content after removal at 12 weeks

After reaching a maximum concentration of 19.0 ng/ml at two weeks after implantation, the formulations eluted readily detectable levels of ivermectin into the bloodstream of cattle for 40 weeks, at which point blood sampling was stopped (Fig. 3). Ivermectin levels above 6 ng/ml, a concentration known to kill 50% of *Anopheles gambiae* within 10 days of feeding exposure [37], was sustained for more than six months. The lowest observed concentration was 3.7 ng/ml at 40 weeks. Figure 3 shows the pharmacokinetic curve for the whole period which is consistent with our previous results in rabbits and pigs (Fig. 4).

### Safety

The maximum ivermectin concentration (C_max_) measured in the plasma of treated calves was 19.3 ng/ml. This value is below the C_max_ achieved with injectable 3.15% and 1% commercial ivermectin veterinary formulations (26 and 114 ng/ml, respectively) that are already widely used. No behavioural side effects (recumbency, depression, ataxia) were observed in the treated calves at any stage of the experiment.

### Mosquito survival after each feeding

**Fig. 5:**
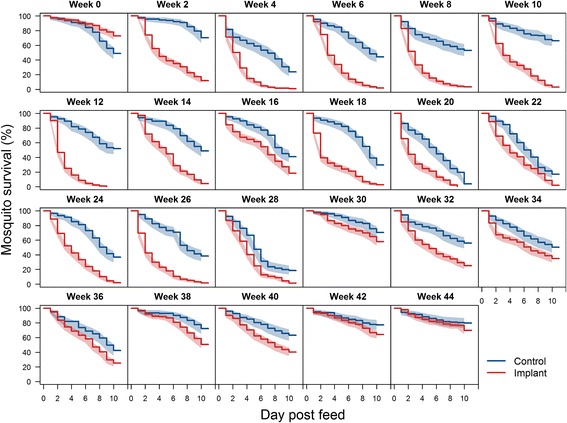
Ten-day survival (and 95% CI) of *Anopheles arabiensis* mosquitoes after feeding on control and treated calves at two-week intervals after implantation (mean *n* = 426, range 327–475). Differences in survival are statistically significant (*P* < 0.05) by log rank test at all time-points

A mean of 436 (range 372–475) fully engorged mosquitoes were followed for ten days after feeding on the calves fortnightly. At this point the control group had a slightly higher 10-day mortality (Log-rank test: *df* = 1*, P* < 0.0001) (Fig. 5). The mortality of mosquitoes feeding after implantation remained elevated throughout the experiment (Log-rank test: *df* = 1, *P* ≤ 0.004) with respect to the control group (Fig. 5).

### Aggregated survival analysis

**Fig. 6:**
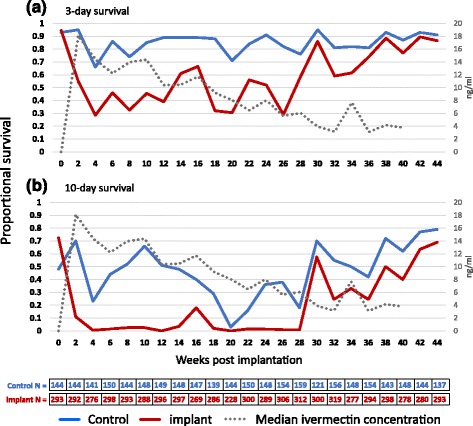
Three-day survival **a** and 10-day survival **b** of *Anopheles arabiensis* mosquitoes after feeding on control and implanted calves every two weeks throughout the experiment. Mean (*n* = 436) fully engorged mosquitoes for each time point, range 372–475. The ivermectin PK is represented as dashed line in reference to the secondary axis

Three-day survival: mosquitoes dying before three days are unlikely to complete the gonotrophic cycle and lay eggs [26], so reductions in 3-day survival can have an impact of large magnitude in overall mosquito densities in the field. While 3-day survival remained high in the controls throughout the experiment (mean 0.85, range 0.66–0.95), it remained consistently lower in the implant group over the first 24 weeks, averaging approximately half that of the controls for the first 25 weeks (mean 0.49, range 0.29–0.67) (Table 2, Fig. 6a).

**Table 2:** Three- and ten-day survival analysis of mosquitoes feeding upon control and implanted calves throughout the study period

		Pre-implant	2 weeks	12 weeks	24 weeks	36 weeks	44 weeks	Mean
3-day survival (proportion)	Control	0.93	0.95	0.89	0.91	0.81	0.91	0.90
	Implant	0.95	0.55	0.39	0.52	0.74	0.87	0.67
	Difference	+0.02 (1%)	-0.40 (42%)	-0.50 (56%)	-0.39 (42%)	-0.07 (8%)	-0.05 (4%)	-0.23 (25%)
10-day survival (proportion)	Control	0.48	0.7	0.51	0.36	0.42	0.79	0.54
	Implant	0.725	0.11	0	0.015	0.245	0.69	0.30
	Difference	+0.25 (51%)	-0.59 (84%)	-0.51 (100%)	-0.34 (95%)	-0.17 (41%)	-0.1 (12%)	-0.25 (46%)

Ten-day survival: mosquitoes dying within 10 days are unlikely to complete the sporogonic cycle and become infectious [26]; this metric directly predicts probability of surviving long enough to incubate malaria parasites through their full sporogonic development, into infectious sporozoites. While in the control group a mean of 40% of mosquitoes survived 10 days or more, only 138 of the 6,016 mosquitoes (2.3%) fed upon implanted calves in the first 26 weeks survived 10 days post-exposure (Table 2, Fig. 6b).

**Fig. 7:**
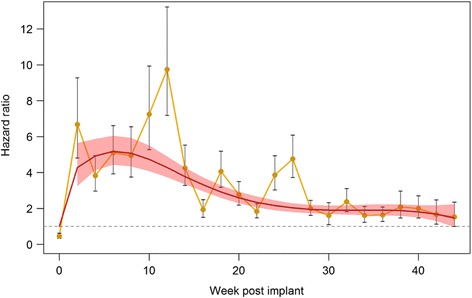
Individual hazard ratios (and 95% CI) for 10-day mosquito mortality after feeding at each time point. Fitted model corresponds to a fourth order polynomial function for weeks 2–44 and is linear from week 0 to 2

Hazard ratios: the individual hazard ratios (and 95% confidence intervals) for 10-day mortality after feeding at each time point as well as the fitting model (fourth order polynomial function) to weeks 2–44 (linear from week 0–2) are presented in Fig. 7 and show an elevated hazard ratio throughout the experiment. All survival data are provided in Additional file 1: Table S1.

## Discussion

We report here a cattle-tailored implantable slow-release formulation of ivermectin, which is capable of sustaining ivermectin concentrations low enough to be safe but high enough to shorten the lifespan of malaria vectors feeding on cattle for six months. The implants are administered through a simple implantation technique using commercially available trocars.

There is currently much interest in mass drug administration to humans to reduce malaria transmission; this will require modification of the dose currently used for NTDs or use of novel long-lasting formulations [27]. In contrast with the administration of ivermectin to humans [38], veterinary applications may offer a potentially easier regulatory pathway to large scale trials and programmatic scale-up. Extensive and reassuring veterinary experience with other formulations that have higher peak blood concentrations (C_max_) in common livestock species should pave the way for formulations like the one described here, which delivers mosquito-killing ivermectin concentrations more steadily over much longer periods. The immediate advantage would the targeted assessment of the many zoophagic malaria vectors that drive residual transmission across the tropics [2, 11, 15], including *Anopheles arabiensis*.

Additionally, this approach provides an opportunity to enhance livestock-based agricultural production, which plays a key role in the livelihood and food security of almost a billion people around the world [39] and obviously depends on sustaining animal health [40]. In sub-Saharan Africa, this could improve the income of 300 million livestock-dependent people [41], by reducing malaria in humans, as well as intestinal helminths and tick-borne diseases in cattle [39, 42]. Emerging tools like the ivermectin implant described here could be promoted and subsidized through agricultural extension systems [43], potentially leveraging novel funding streams and intersectoral collaborations.

Regarding long-lasting formulations in livestock, one key challenge is the induction of ivermectin resistance in intestinal helminths, which is already a serious issue in several parts of the world [44], or even among mosquitoes themselves. In the latter case, proven reduction of mosquito fertility after feeding on sub-lethal ivermectin concentrations could help delay the appearance of eventual ivermectin resistance [45]. Additionally, veterinary ivermectin is not envisioned as a stand-alone tool, rather as complementary strategy to the home-based standard of care with LLINs/IRS, a combination that is predicted to be synergistic [46] or as a potential resistance management tool given its different class allowing for combination of different delivery methods rather than mixture of insecticides. Concerns regarding veterinary helminths and non-target organisms [47] could be assessed with drug combinations, refugia [48] or different endectocides.

Although this evaluation does confirm the potential of this novel formulation, the study also has important limitations of that merit consideration going forward. First, many of the most attractive secondary outcomes that could confirm safety or motivate uptake by livestock owners were not recorded. Although the calves were physically examined daily by a herdsman and monthly by a qualified veterinarian, we did not include any formal assessment of growth or health. Also, defining the withdrawal period for this new formulation will require determination of tissue residual concentrations and against potential dietary exposure [49].

Secondly, mosquitoes were kept in a semi-field system with near ambient conditions of temperature, humidity and air flow, which has both advantages and disadvantages. While the survival estimates obtained here may be more representative of field conditions than other studies in fully-controlled laboratory environments, such exposure to near-natural variations in ambient weather conditions may also underpin some of survival rate fluctuations observed from one fortnight to another (Fig. 6). Thirdly, and perhaps most important, we used only a very limited number of healthy calves for this experiment, so further studies with greater replication and statistical power will be needed in a wider diversity of settings and livestock species including those affected by intestinal parasites of veterinary importance.

Nevertheless, the pharmacokinetic observations are consistent with previous results with this formulation in rabbits [31], dogs [34] and pigs [32] (Fig. 4). Given this prolonged drug release at effective levels, field trials with this or similar formulations will need to monitor the vector species breakdown to ensure the number of anthropophagic vectors does not increase as a result of selective pressure on zoophagic vectors, even if modelling suggest synergism with LLINs [46].

## Conclusions

Despite the study limitations described immediately above, it does appear that incremental impact upon residual malaria transmission by zoophagic vectors could be obtained through high area-wide coverage of livestock with this novel ivermectin formulation, or with other formulations capable of similarly stable and sustained efficacy over several months.

## Additional file

Additional file 1: **Table S1.** Survival analysis in all mosquito groups throughout the experiment. (XLSX 11 kb)

## Abbreviations

C_max_: Maximum drug concentration
GTS: Global Technical Strategy
HPLC: High-performance liquid chromatography
IRS: Indoor residual spraying
LLINs: Long-lasting insecticidal nets
NTDs: Neglected tropical diseases
